# Supplementary material for: The effects of genital myiasis on the diversity of the vaginal microbiota in female Bactrian camels
Source: BMC Vet Res. 2022 Mar 5;18:87. doi: 10.1186/s12917-022-03189-5 (PMC8897907; doi:10.1186/s12917-022-03189-5)
Supplement: Supplementary file 5 — Additional file 5. [file 12917_2022_3189_MOESM5_ESM.zip › MPL201709200_16s_yy/Treat1/css/js_css/jsPDF-master.zip!/jsPDF-master/examples/html2pdf/total_mess.html]

Html2Pdf


# Heading one

## Header two

### Header three

#### Header four

##### Header five

###### Header six

This is a copy of one of the sample pages from the WordPress theme development test content. I found it handy to keep a copy of this for building sites that aren’t using WordPress. 99% of the credit goes to them, I’m just hosting it in a handy place in case it’s useful to anyone other than me.

## Blockquote Tests

Blockquote:

> Here’s a one line quote.

This part isn’t quoted. Here’s a longer quote:

> I have learned, that if one advances confidently in the direction of his dreams, and endeavors to live the life he has imagined, he will meet with a success unexpected in common hours.
>
> Henry David Thoreau

And some trailing text.

## Table Layout Test

| Title | Views |  |
| --- | --- | --- |
| About Test User | 1 | More |
| 260 | 1 | More |
| Archives | 1 | More |
| 235 | 1 | More |

## List Type Tests

### Definition List

Definition List Title
:   This is a definition list division.

Definition
:   An exact statement or description of the nature, scope, or meaning of something: *our definition of what constitutes poetry.*

Gallery
:   A feature introduced with WordPress 2.5, that is specifically an exposition of images attached to a post. In that same vein, an upload is “attached to a post” when you upload it while editing a post.

Gravatar
:   A globally recognized avatar (a graphic image or picture that represents a user). A gravatar is associated with an email address, and is maintained by the Gravatar.com service. Using this service, a blog owner can configure their blog so that a user’s gravatar is displayed along with their comments.

### Unordered List (Nested)

- List item one
  - List item one
    - List item one
    - List item two
    - List item three
    - List item four
  - List item two
  - List item three
  - List item four
- List item two
- List item three
- List item four

### Ordered List

1. List item one
   1. List item one
      1. List item one
      2. List item two
      3. List item three
      4. List item four
   2. List item two
   3. List item three
   4. List item four
2. List item two
3. List item three
4. List item four

## HTML Element Tag Tests

All other HTML tags listed in the FAQ:

Here is the address for Automattic, using the
 
tag:

355 1st Street Suite 202  
 San Francisco, CA 94105  
 United States

This is an example of an   (otherwise known as a link). This abbr. is an example of an  tag in the middle of a sentence. Here is a TLA showing off the   tag. What, you want to see some over-sized text using the   tag? Can you cite a reference for that, using the   tag? Have you noticed that all of the tag names are displayed `in code-form`, using the  `tag? Similarly, I could
emulate keyboard text
, using the


text tag, or
emulate teletype text
using the


tag.`

Oh no! I wrote something incorrectly.
~~I’d better delete it~~
, using the
 
tag. I could alternately strike something out using the
 
tag, or strike something out using the
 
tag. *So many choices*, which I emphasize using the
 
tag. Just to clarify,
this is some inserted text
, that I’ll highlight using the
 
tag.

Need to display completely unformatted text, such as a large block of code? Use the
 ```` ```
			
``` ```` 
tag, which lets you display:

```
#container {
    float: left;
    margin: 0 -240px 0 0;
    width: 100%;
}
```

Want to quote the WordPress tagline "Code is Poetry"? Use the
 `"
"`
tag to add quotes around it. **This is strong text** (otherwise known as bold), using the
 
tag.

Need to write H_2_O or E = MC^2^? You may find great use for _subscripting_ text using the
 
tag, or for ^superscripting^ text using the
 
tag. Need to call out a
variable
? Use the
 
tag.

## Div and Span Tests

Lorem ipsum dolor sit amet, consectetuer adipiscing elit.

**This is a div with “myclass” class, inside of another div, using the  tag.**

Sed odio nibh, tincidunt adipiscing, pretium nec, tincidunt id, enim. Fusce scelerisque nunc vitae nisl.

Lorem ipsum dolor sit amet, consectetuer adipiscing elit. **This is a span inside of a paragraph, using the   tag.**  Sed odio nibh, tincidunt adipiscing, pretium nec, tincidunt id, enim. Fusce scelerisque nunc vitae nisl.
